# Role of Sphingolipids in the Pathobiology of Lung Inflammation

**DOI:** 10.1155/2015/487508

**Published:** 2015-12-03

**Authors:** Riccardo Ghidoni, Anna Caretti, Paola Signorelli

**Affiliations:** Department of Health Sciences, University of Milan, San Paolo Hospital Medical School, Via Di Rudinì 8, 20142 Milan, Italy

## Abstract

Sphingolipid bioactivities in the respiratory airways and the roles of the proteins that handle them have been extensively investigated. Gas or inhaled particles or microorganisms come into contact with mucus components, epithelial cells, blood barrier, and immune surveillance within the airways. Lung structure and functionality rely on a complex interplay of polar and hydrophobic structures forming the surfactant layer and governing external-internal exchanges, such as glycerol-phospholipids sphingolipids and proteins. Sphingolipids act as important signaling mediators involved in the control of cell survival and stress response, as well as secreted molecules endowed with inflammation-regulatory activities. Most successful respiratory infection and injuries evolve in the alveolar compartment, the critical lung functional unit involved in gas exchange. Sphingolipid altered metabolism in this compartment is closely related to inflammatory reaction and ceramide increase, in particular, favors the switch to pathological hyperinflammation. This short review explores a few mechanisms underlying sphingolipid involvement in the healthy lung (surfactant production and endothelial barrier maintenance) and in a selection of lung pathologies in which the impact of sphingolipid synthesis and metabolism is most apparent, such as acute lung injury, or chronic pathologies such as cystic fibrosis and chronic obstructive pulmonary disease.

## 1. A Brief Overview on Sphingolipids within the Lung Environment

The interest in sphingolipid presence and bioactivities in the respiratory airways has produced a steady number of reports since the 1970s. However, a host of publications in the last few years have provided an increasingly detailed picture of the role played in the lungs by this class of lipids and by the proteins that handle them. As vital respiratory organs that mediate air-blood gas exchanges, lungs must undergo delicate and tightly controlled developmental transitions. Antenatally, a 20-week human fetus displays lungs that have branched to generate all airways, but it is not before ~28 weeks of gestation that alveolarization begins from primordial saccular structures and type I alveolar cells differentiate from the cuboidal epithelium. Concomitantly, at this stage endothelial cells shape the alveolar capillary bed and type II alveolar cells appear, to demarcate alveolar septal junctions. Type II cells start producing surfactant, which accumulates to increasing concentrations by term. The initiation of autonomous ventilation at birth represents a dramatic switch in postnatal lung function. While throughout gestation a chloride-ion driven liquid secretion creates a positive pressure that distends the lungs and stimulates growth, a sudden reversal from net secretion to net adsorption takes place at birth under the effect of O_2_ and hormones (epinephrine, glucocorticoids, and thyroid hormones), enabling the rapid elimination of lung liquid. From this moment on, lung lumen will maintain a low-level chloride-ion based liquid secretion to generate a surface liquid layer, known as surfactant and formed by specific secreted lipids and proteins, and a robust absorptive capacity will prevent alveolar flooding and edema.

Equally important, being permanently exposed to inhaled particles and microorganisms from birth, pulmonary immunity must be tuned to effectively dispose of them, while minimizing immunopathology to preserve appropriate gas exchange. Thus, the first-line lung defenses, prior to immunity, are based on mechanical weapons including cilia, mucus, and the cough reflex, which concur to prevent pathogen access to the lower airways and in so doing avoid an overt inflammatory response. This is one of the major reasons why lungs are particularly sensitive to the sphingolipid (and other lipids) metabolism equilibrium: pulmonary physiology relies on lipids for important extracellular activity ensured by surfactant and consisting of a sphingolipid/glycerolipid network. Indeed, most successful respiratory pathogens have evolved the ability to gain access to the lower airways in the alveolar compartment, the critical lung functional unit involved in gas exchange. Inherited conditions such as cystic fibrosis are prone to lung infection, partly as a consequence of a compromised mechanical clearance. Upon infection or in sterile inflammatory conditions, adjoining epithelial and endothelial layers in the alveoli, which with their fused basal lamina form the next leakproof barrier against microbes, must become temporarily permeable to allow leukocyte migration. This way, bone marrow derived macrophages, neutrophils, and dendritic cells can enter the extra-epithelial space where they meet a resident population of yolk sac-derived macrophages. A complex cross talk between specific cell populations (immune cells, epithelial cells, endothelial cells, stromal cells, and platelets), mediators, and coagulation and complement cascades will orchestrate the immune response and inflammation from start to resolution. However, disruption of alveolar integrity may occur in aging and in a number of lung diseases.

How do sphingolipids fit into the above sketch? As in most other tissues and organs, in the lung too, sphingolipids play a crucial role as signaling molecules as well as components of membranes and extracellular fluids, in both normal development/functioning and pathological settings involving inflammation. A lung-specific action is the one involving sphingolipids as both minor structural components of lipid-protein surfactant and regulators of its synthesis and release. This short review explores a few mechanisms underlying sphingolipid involvement in the healthy lung (surfactant production and endothelial barrier maintenance) and in a selection of lung pathologies where the impact of sphingolipid metabolites is most apparent. Drawing upon recent evidence from studies in humans and in animal models and* in vitro*, including those in our laboratory, we attempt to highlight in particular the reciprocal controls intertwining sphingolipid metabolism with that of other bioactive lipids (i.e., glycerophospholipids) and the vicious circle linking sustained cellular stress and the imbalance of* de novo* sphingolipid synthesis, as a significant mechanism acting in favor of tissue injury and against inflammation resolution in inflammatory lung diseases. Further, current therapeutic perspectives in using compounds that target the sphingolipid pathway for countering development of lung injury will be discussed. For a more extensive coverage of additional lung pathologies and the role of sphingolipids therein, the reader is referred to other recent reviews [1–4]. In addition, here we shall take for granted the general notions about sphingolipid biosynthesis, transport, and breakdown, which have been extensively treated in many reviews, and shall just recall those that are relevant to our narrative.

## 2. Sphingolipid Synthesis during Pulmonary Functional Maturation

**Figure 1:**
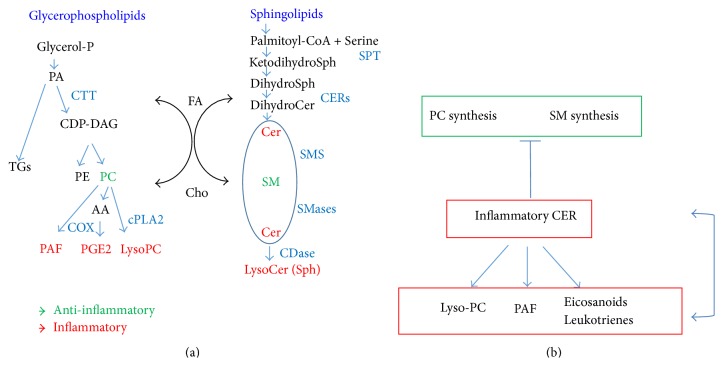
Sphingolipids metabolites form a network with other inflammatory lipids in the lung. (a) Intracellular and secretory lipids regulating inflammation in mucus and mucosa. Red: inflammatory lipids and major controlled enzymatic activities. Green: anti-inflammatory lipids and major controlled enzymatic activities. P, phosphate; FA, fatty acids, Cho, choline; CDP, citidyl diphosphosphate; DAG, diacylglycerol; TGs, triglycerides; PE, phosphatidyl ethanol ammine; PC, phosphatidyl choline; PAF, platelet activating factor; AA, arachidonic acid; PGE2, prostaglandins E2; Sph, sphingosine; Cer, ceramide, SM, sphingomyelin; CTT, CTP: Phosphocholine Cytidyl-Transferase; SMase, Sphingomyelinase, SMS, Sphingomyelin Synthase, and CERS, Ceramide Synthases; SPT, Serine Palmitoyl Transferase; cPLA2, Cytosolic Phospholipase 2. (b) Ceramide accumulation controls other inflammatory and anti-inflammatory lipids. Red: inflammatory lipids and major controlled enzymatic activities. Green: anti-inflammatory lipids and major controlled enzymatic activities.

About five decades ago, we learned of the presence of a layer of surface lipids, with structural and active signaling roles, consisting of lecithin, dipalmitoyl-lecithin, sphingomyelin, phosphatidyl ethanolamine, phosphatidylinositol, phosphatidylglycerol, cholesterol, and small amounts of other lipids, along with proteins of 18 and 36 kDa (termed surfactant proteins), beginning to form at 28 days of gestation [5, 6]. This notion led to the use of a lecithin/sphingomyelin (L/S) ratio, from amniotic fluids, in the clinical diagnosis of fetal lung maturity [7] and in the prediction of neonatal respiratory distress syndrome RDS caused by an insufficient amount of pulmonary surfactant [8, 9]. An L/S ratio less than 2.0 indicates a potential risk of RDS. The risk is nearly 75–80% when the L/S ratio is 1.5. Experiments conducted in rats demonstrated that the prenatal development of this surface lipid activity sees sequentially ordered changes in components, including glycerophospholipids (phosphatidylserine and phosphatidylinositol) [10] together with the sphingolipid sphingomyelin [11]. Similarly, sequential changes in the tissue expression of these lipid-related enzymes were shown, identified in the microsomal fraction of respiratory epithelia and in the alveolar lavage. During the third trimester, the fetal lung synthesizes primarily sphingomyelin, and the majority of stored glycogen is converted to fatty acids and then to surfactant lipids. The enzymes responsible for the biosynthesis of phosphatidyl glycerolipids (CTP: Phosphatidate Cytidylyl Transferase and CDP-Diacylglycerol: Glycerolphosphate Phosphatidyl Transferase and Phosphatidyl Glycerol Phosphate Phosphatase) demonstrated a coordinate increase in activity in fetal rat lung at term when the demand for pulmonary surfactant increases. A fall in one phospholipid is accompanied by an increase in another. The same phenomenon is observed among phospholipids in human amniotic fluid, suggesting a similar development and further confirming the continuity between fetal lung secretions and the amniotic fluid. At birth, the expression of the major enzymes involved in phosphatidyl choline synthesis (CTP and CDP) as well as the expression of Serine Palmitoyl Transferase and Sphingomyelin Synthase further increases, respectively, in type I [10] and type II alveolar cells [11]. Ceramide Synthase 5 is the predominant isoform detected in lung epithelia and its expression is also upregulated. Concomitantly synthesized at ER [12], phosphatidylcholine and sphingomyelin are both secreted in the surfactant, whereas lung tissue, other than surfactant, contains only small amounts of these phospholipids. It is noteworthy that sphingolipid and glycerolipid metabolisms overlap at the enzymatic step where ceramide competes with diacylglycerol for the phosphocholine (deriving from CDP-choline or phosphatidylcholine) to give rise to sphingomyelin (Figure 1). In adult life, enzyme activity reaches a plateau, and the ratio between the key enzymes of the two pathways, Serine Palmitoyl Transferase over Glycerol 3-phosphate Acyl Transferase, is significantly higher in microsomal lung (and pancreas) than in most other adult rat tissues; accordingly, the percentage of sphingomyelin is higher in the total phospholipid content in these fractions [13].

Fetal lung maturation allows protection from maternal infection, but this maturation process can be altered by pathogens [14]. Surfactant components generally decrease harmful inflammatory responses [15]. Preterm labor-inducing inflammatory ligands (interleukin-1 or lipopolysaccharide) cause a robust induction of the surfactant complex in order to lower the risk of respiratory distress syndrome (RDS). Data from clinical studies suggest that surfactant can be used successfully in neonates with congenital pneumonia, due to a comprehensive contribution by surfactant to host defense from pathogens [16, 17]. However, such trials obtained mixed results and much evidence in animal studies demonstrates that surfactant therapy may also enhance inflammation and reduce the ability of macrophages to clear pathogens [18–20], raising the hypothesis that surfactant physiologic activity relies on a complex equilibrium, whose alteration drives a pathological setting. Thus, an unbalanced sphingolipid metabolism impact on surfactant composition and pulmonary function as well as pharmacological intervention aimed at regulating sphingolipids mediators can promote or block surfactant production. A more extensive comprehension of sphingolipid moiety of surfactant would help to identify therapies or adjuvants for most lung diseases.

## 3. A First Glance at the Small Picture: Sphingolipid Molecules Take Part in Airway Cell Signaling in Inflammatory Responses

Before dealing with pulmonary disease, we should review better the evidence collected for sphingolipid signaling in lung-derived cell cultures under pathologic stress. The very first evidence of sphingolipid involvement in lung inflammation concerns the production of autoantibodies against glycosphingolipids (aGM1 and GM1b) in response to pulmonary infection with* Mycoplasma pneumonia* [21]. Damaged or regenerating respiratory epithelial cells, typical of cystic fibrosis or lungs suffering from emphysema, exhibit increased expression of receptors for sialylated glycosphingolipids [22], which are recognized by Gram-positive and Gram-negative organisms. Upon engagement, these receptors activate the inflammatory signaling cascades of the acute innate phase [23]. Moreover, alveolar macrophages undergo maturation steps, showing surface expression of aGM1 [24, 25].

In addition to infection, it is possible to reproduce lung inflammation, in an* in vitro* setting, by stressing cells with oxidative agents (e.g., hydrogen peroxide), which represent the by-products of inflammatory damage. Alternatively, cells can be stressed directly with inflammatory mediators or by blocking trophic and prosurvival stimuli, which are dramatically reduced in pulmonary low oxygen pathological conditions (i.e., by inhibiting Vascular Endothelium Growth Factor (VEGF) receptor signaling). Most of the* in vitro* evidence suggests that ceramide can be pharmacologically targeted to reduce reactive oxygen and nitrogen species and inflammatory damage in airway cells. In 2000, Chan and coauthors demonstrated, at a molecular level in the human airway epithelial cell line (HAE), the dependency of hydrogen peroxide-induced apoptosis on ceramide generation following the activation of the glutathione-sensitive neutral Sphingomyelinase [26, 27]. A few years later, Goldkorn and his group demonstrated that hydrogen peroxide upregulates neutral Sphingomyelinase 2 and consequently increases ceramide levels in human airway epithelial cells [28, 29]. IL-8 plays a pivotal role in lung injury, serving as a recruiter for neutrophils. Neutrophil invasion causes a massive release of oxygen radicals, proteases, and other toxic moieties, responsible for the subsequent tissue destruction and loss of barrier function, leading to pulmonary edema, intrapulmonary shunt, and hypoxemia hallmarks. IL-8 neutralization can be envisaged as a therapeutic approach [30]. Glutathione inhibition of neutral Sphingomyelinase counteracts part of the oxidative stress-induced signaling (p38 activation, inhibition/degradation of the bound phosphatase calcineurin) that sustains IL-8 transcriptional activation [31]. Apart from reactive oxygen species, such as hydrogen peroxide, reactive nitrogen species (RNS) are involved in the pathophysiology of inflammatory lung diseases. NO exposure (via NO donors) was able to induce ceramide accumulation but not apoptosis in airway epithelial cells, by stimulating Ceramide Synthases. This accumulation was inhibited by fumonisin B1 (inhibitor of Ceramide Synthases) [32]. According to the notion that different pools of ceramide can be raised by different stress inducers and take part in specific responses [33], not only neutral Sphingomyelinase but also the activity of Ceramide Synthases is therefore modulated in airway epithelia inflammation. The combined presence of high levels of NO and superoxides generated peroxynitrite (ONOO(−)), which is responsible not only for the ceramide increase but also for apoptosis induction. Such apoptosis was prevented by silencing acid Sphingomyelinase [34].

Taken together, these findings support the hypothesis that the inflammatory stress in airway epithelia, driven by oxygen and nitrogen oxidative species, modulates different pools of ceramide, possibly involving its* de novo* synthesis until the stress is tolerated and evolving to Sphingomyelinases activation to induce apoptosis.

Ceramide can be deacylated by Ceramidases to give rise to the toxic sphingosine, which can be immediately phosphorylated to sphingosine-1-phosphate. Among all the sphingolipids, sphingosine-1-phosphate is a minor species in terms of intracellular concentration but is endowed with potent proliferative and prosurvival activity. The high level of plasma sphingosine-1-phosphate, bound either to albumen or to lipoproteins, has important homeostatic functions in the maintenance of vascular integrity [35] and its gradient is crucial for immune cell trafficking during inflammatory reactions [36]. The role of sphingosine-1-phosphate in inflammation is partially controversial. Although* in vivo* it exerts mainly an anti-inflammatory role (discussed in the following sections), a few reports demonstrate proinflammatory activities of this lipid mediator. H441 lung epithelial cell treatment with exogenous sphingosine-1-phosphate induced an increase in IL-8 mRNA and its secretion. TNF*α* can also activate Sphingosine Kinase [37, 38], and sphingosine-1-phosphate leads to IL-8 gene expression* via* ERK and p38 MAPK activation and increased AP-1 inflammatory transcriptional activity in alveolar macrophages [39]. Thus sphingosine-1-phosphate recapitulates the action previously ascribed to ceramide and possibly exerted by sphingosine-1-phosphate. A putative explanation may be that sphingosine-1-phosphate, either supportive or alternative to ceramide, could exert different actions not only on cell culture treatment versus* in vivo* but also in physiological and protective inflammatory responses [26], as opposed to pathological inflammation.

Human pulmonary artery endothelial cells effectively utilize exogenous sphingosine-1-phosphate as a prosurvival and permeability regulator, via extracellular conversion to sphingosine by Lipid Phosphate Phosphatase-1 and uptake of sphingosine followed by intracellular phosphorylation by Sphingosine Kinase-1 [40, 41]. VEGF receptor is engaged by a trophic factor required for the survival of endothelial cells and abundantly expressed in the lung [42]. The inhibition of VEGF receptor initiates apoptosis and alveolar destruction, morphologically resembling emphysema, and it is concomitant to an increased ceramide synthesis in the alveolus [43, 44]. FTY720, a sphingosine analogue which can be phosphorylated [45], acts as a downregulator of sphingosine-1-phosphate receptor 1 by inducing its ubiquitination, internalization, and degradation [46, 47], but it was also proved to directly inhibit Ceramide Synthases activity in human pulmonary artery endothelial cells [48]. Consequently FTY720 may at the same time target ceramide synthesis and sphingosine-1-phosphate signaling, which is downstream of ceramide catabolism. A contrasting but interesting report supports the hypothesis of a complex and fine regulation of ceramide signaling pools in which TNF*α*-induced ceramide in lung epithelial cells is necessary to downregulate IL-8 synthesis. In line with previously reported ceramide activation of the Protein Phosphatase 2A (PP2A) [49] and ceramide impairment of the binding of noncompetitive biological inhibitors of PP2A [50], Cornell et al. reported that TNF*α*-induced ceramide, in respiratory epithelial cells, activates PP2A, which is responsible for dephosphorylation/inactivation of MAPK pathways (JNK, p38, and ERK) and consequently for the inhibition of their downstream NF-kB promoter activity. This would explain the downmodulation of the IL-8 transcription. The authors showed that both desipramine (inhibitor of acid Sphingomyelinase) and fumonisin B1 pretreatments (for a few hours) block the initial ceramide increase upon TNF*α* receptor engagement, indicating a double pathway leading to ceramide accumulation from* de novo* synthesis and from the sphingomyelin cycle. This initial ceramide wave is, in their hypothesis, important in a feedback regulation of inflammatory signaling and its absence enhances IL-8 release [51]. These apparently contrasting data, suggesting a ceramide role of “inflammation homeostasis keeper,” can be explained if we consider an overall picture of a physiological inflammatory setting versus chronic inflammatory pathology. Acute stimulation of early inflammatory inducers, such as TNF*α*, may trigger a controlled remodeling of sphingolipid mediators, during which ceramide can be driven into metabolic transformation such as the sphingomyelin cycle or the phosphorylation to ceramide-1-phosphate. The latter, involved in inflammation with contrasting evidence [52], was shown to mediate TNF*α*-induced IL-10 production, thus representing the missing link that relates proinflammatory to anti-inflammatory/physiological inflammation resolution response [52, 53].

In this line of thinking, an initial ceramide-driven reaction to inflammation could be aimed at inflammation resolution, but it is conceivable that deregulation of ceramide metabolism enzymes, due to excessive stimuli and stress, can become an effector arm that easily pushes the whole inflammatory machine to collapse into pathological chronic inflammatory signaling.

## 4. Sphingolipid Metabolites Form a Network with Other Inflammatory Lipids in the Lung

PAF is a potent lipid mediator which is involved in asthma, sepsis, and acute lung injury, responsible for vasoconstriction, bronchoconstriction, vascular permeability, and pulmonary and extra-pulmonary edema formation. PAF-induced increase in vascular permeability is mediated by PGE2 and ceramide, derived from acid Sphingomyelinase activation, in endothelial cells [54, 55]. On the other side, PAF-induced activation/maturation of macrophages during inflammation was related to* de novo* ceramide synthesis induction [56]. Moreover, the Sphingomyelinase activation and ceramide accumulation promote Cyclooxygenase 2 expression and its release of PGE2 [57].

Being a major surfactant component, phosphatidyl choline is largely used in clinical therapy as a potent anti-inflammatory agent for intestinal mucosa protection [58, 59]. In airway epithelia cells, the interrelation of sphingolipids and glycerolipids metabolism was assessed by demonstrating that cytokine-induced catabolism of sphingomyelin, related to the inflammatory ceramide release, and the inhibition of the anti-inflammatory phosphatidyl choline synthesis are directly dependent on one another, suggesting that a complex program of lipid adjustment is targeted to initiate inflammatory response. TNF*α*-derived ceramide and sphingosine inhibit Phosphatidyl-Choline: Ceramide Phosphocholine Transferase (Sphingomyelin Synthase) [60, 61], blocking the “consumption” of phosphatidyl-choline to form sphingomyelin and diacylglycerol. At the same time, in H441 cells, TNF*α*-induced ceramide reduces phosphatidyl choline synthesis, probably because of its inhibition of CTP: Phosphocholine Cytidylyl-Transferase (CTT), which is the rate-limiting enzymatic step in* de novo* phosphatidyl choline synthesis [60, 61]. In this view, the inflammatory stimulus TNF*α* raises ceramide release,* via* Sphingomyelinase activation. Ceramide activates cytosolic PLA2, thus increasing lysophosphatidyl choline, which on the contrary is a proinflammatory mediator [62] and inhibits the phosphatidyl choline synthesis (CTT activity). The arachidonic acid, released by cPLA2 upon lysophosphatidyl choline formation, stimulates the synthesis of leukotrienes, which in turn raise intracellular Ca^2+^ levels and complete the activation of cPLA_2_ [60], as well as lysosomal phospholipases [39] (Figure 1). Interestingly, the overexpression of Ceramide Synthase 5, the predominant Ceramide Synthases isoform detected in lung epithelia, also reduced phosphatidyl choline synthesis, but maximal inhibition was achieved when Ceramide Synthase 5 was coexpressed with a plasmid encoding a neutral Sphingomyelinase, enhancing sphingomyelin hydrolysis [63]. Thus, the modulation of sphingolipid metabolism drives the formation of glycerolipids inflammatory molecules as well as the eicosanoids family of inflammatory lipids. Altered surfactant is undoubtedly related to inflammatory stress within the lungs. Inflammatory cytokines regulate the alveolar pool of sphingomyelin. Sphingomyelin hydrolysis, induced upon inflammation, causes a twofold increase in the amount of surfactant-associated ceramide, tending to decrease the sphingomyelin mass, thus impairing the biophysical properties of the alveolar surfactant film [64]. Moreover, ceramide can interfere with surfactant production and release. All these lipids and surfactant components are secreted by type II pneumocytes by regulated exocytosis of secretory vesicles, termed lamellar bodies. The fusion of lamellar bodies with the plasma membrane is inhibited by treatment with the ceramide analogue C2-ceramide, which inhibits phospholipase D activity [65]. Moreover, in H441 airway epithelial cells, ceramide decreased SP-B surfactant production. This was shown to occur by a unique ability of ceramide to bind to a region located within the −233/−80 bp region of human SP-B promoter. Ceramide binding was shown to reduce the transactivation capability of thyroid transcription factor 1 (TTF-1/Nkx2.1), a key factor for SP-B promoter activity [66]. An overall view of sphingolipid interaction with other lipids and of their inflammatory and anti-inflammatory activities is provided in Figures 1(a) and 1(b).

## 5. Sphingolipid in Pulmonary Inflammatory Pathologies: Acute and Chronic Inflammation Are Sustained by Sphingolipid Mediators

Pathological inflammation in the lung involves surfactant and mucus production, epithelial cell reaction, endothelial permeability, immune response, parenchyma, and matrix damage. The major outputs are leukocyte infiltrate, releasing damaging molecules such as radicals and proteases; tissue edemas and fibrosis remodeling; small terminal airway damage (bronchiolar loss) with low oxygen-hypoxia induction; capillary damage and hypertension; necrosis and emphysema. In the following paragraphs we will review the involvement of sphingolipid mediators and the adjustments of sphingolipid metabolism in the setting of pulmonary inflammation, focusing only on acute lung injury and on two major chronic diseases, namely, cystic fibrosis and chronic and obstructive pulmonary disease.

### 5.1. Pulmonary Inflammation and Acute Respiratory Distress Syndrome (ARDS)

Pulmonary inflammation, generally known as Adult Respiratory Distress Syndrome (ARDS), occurs in individuals who sustain systemic or localized insults (sepsis, aspiration of toxins, emboli, circulatory collapse, and metabolic neurological hematological disorders) that cause diffuse lung injury. Acute lung injury (ALI) is the most severe form. The major event is fluid leaking into the lungs from damaged capillaries (edema). Disappointing results from therapeutic approaches, targeting known involved mediators such as TNF*α*, PAF, or PGEs, suggest that several parallel and interacting mechanisms are involved. Sphingolipid altered metabolism was shown to take part in this pathological process.

As early as 1985, Merrill and his collaborators noticed a significant decrease in sphingomyelin in the lung microsomal fraction of rats maintained in elevated versus normal oxygen levels [67]; later, alteration in oxygen supply (hyperoxia) was shown to be a potent cause of ceramide accumulation and of lung injury and inflammation [68–70]. This early evidence shed light on the hypothesis, largely validated later on, that sphingolipids are modulated during inflammatory processes relating to airway oxygenation and may actively take part in inflammation responses.

Besides surfactant phospholipids, bronchoalveolar lavage from ARDS patients contains a significant amount of ceramides and glycosphingolipids (lactosyl ceramides and paraglobosides), appearing during lung injury, that are present just in traces in healthy people [71]. The glycosphingolipids take part in damage because they are able to inhibit the surfactant system* in vitro* by increasing surfactant tension obtainable at minimum bubble size [71]. Ceramide derivatives are markedly elevated in bronchoalveolar lavage fluid of patients with ARDS [71] and plasma ceramide levels correlate with mortality [72].

Göggell and coworkers extensively analyzed the role of ceramide in lung edema, demonstrating that ceramide accumulation in lung tissue and fluids, upon acid Sphingomyelinase increased activity, is an important cause of edema formation [73]. Mice treated with PAF developed pulmonary edema which was reduced by ~50% in acid Sphingomyelinase deficient animals. A further decrease was obtained if deficient animals were treated with acetylsalicylic acid, indicating a common involvement of acid Sphingomyelinase and cyclooxygenase in the pathogenesis of pulmonary edema. Moreover, the perfusion of rat lungs with TNF*α* or PAF rapidly induced an increased ceramide concentration in the alveolar fluid, suggesting increased activity of extracellular acid Sphingomyelinases. Antisera against ceramide as well as the pharmacological inhibitors of the membrane cycle sphingomyelin/ceramide (xanthogenate D609 for Sphingomyelin Synthase and imipramine for acid Sphingomyelinase) prevented PAF-triggered pulmonary edema. Nonetheless, inhibition of ceramide release from the plasma membrane had no effect on other PAF actions, such as reduction in pulmonary vasoconstriction and bronchoconstriction. The authors exclude that ceramide may derive from a biosynthesis pathway because fumonisin B1, an inhibitor of Ceramide Synthase, had no effect on pulmonary edema. In a neonatal piglet model of airway inflammation, induced upon repeated lavages, surfactant plus desipramine administration prevented edema, inflammatory marker upregulation, leukocyte alveolar influx, and increase in ceramide content. Similar results were obtained in acid Sphingomyelinase deficient mice treated with surfactant, which were protected from inflammation unlike Sphingomyelinase-expressing control animals [74].

Acute pulmonary injury can be modeled by bleomycin-induced inflammation and fibrosis in mice. Such pathological consequences correlated with the rapid activation of acid Sphingomyelinase, and injury was markedly attenuated in the absence of the enzyme (knockout mice). Along with the elevated acid Sphingomyelinase activity, there was an increase in acid ceramidase activity, which was sustained for up to 14 days after bleomycin treatment, suggesting a possible bioactivity of the induced accumulation of sphingoid back-bone mediators such as sphingosine and sphingosine-1-phosphate. Consistently with this hypothesis, bleomycin treatment induced acid Sphingomyelinase and acid Ceramidase increased activity and accumulation of sphingosine-1-phosphate in NIH3T3 fibroblasts [75]. Nonetheless, the formation of sphingosine-1-phosphate was able to counteract the inflammation-induced endothelial permeability [76, 77] and pharmacological use of its analogues (FTY720, s-FTY720-phosphonate, and SEW2871) was proposed as a therapeutic approach in ALI [78].

Although these data suggest a key role for acid Sphingomyelinase activation and ceramide formation to cause edema, studies in human airway epithelial cells have shown that neutral Sphingomyelinase, but not acid Sphingomyelinase, is activated to induce cell death by inflammatory stress, such as cigarette smoke [28]. Hyperinflammation, such as LPS treatment, is associated with induced suppression of spontaneous neutrophil apoptosis, whose peculiarity is to provide for their own suppression, being short-lived differentiated cells. A massive and uncontrolled presence of neutrophils in the lung contributes to inflammation, thus entering a pathological vicious cycle. Lin and colleagues demonstrated significantly higher ceramide levels in alveolar neutrophils from ARDS patients than in those from healthy subjects, indicating an association between inflammatory granulocytes and increased ceramide level, specifically in the alveolar areas [79]. These authors showed that Sph-24 (neutral Sphingomyelinase inhibitor) and SKI-II (Sphingosine Kinase I inhibitor, triggering the enzyme lysosomal degradation [80]) antagonized the antiapoptotic effect of LPS [79]. Interestingly the acid Sphingomyelinase inhibitor CHL (inducing the enzymes lysosomal degradation [81]) had no effect on the regulation of neutrophil apoptosis in response to LPS stimulation.

Thus ceramide is one of the required triggers of inflammation, endothelial leaking, and edema in pulmonary injury; the acute inflammatory stress would cause a burst in sphingolipid catabolism, both at the acidic lysosomal compartment and at the plasma membrane neutral compartment, thus involving multiple inflammatory signaling. Sphingosine and sphingosine-1-phosphate accumulation may derive from lysosomal-related ceramide formation and possibly contribute to inflammation with diverse effects depending on the cell type and sphingosine-1-phosphate increase may impair neutrophils spontaneous apoptosis. The underlying idea, stemming from these reports and suggested by von Bismarck and colleagues, is that it may be possible to create a “fortified surfactant preparation,” enriched in anti-inflammatory lipids and/or enzyme inhibitors (i.e., Sphingomyelinases inhibitors), with therapeutic activity against newborn lung inflammation.

### 5.2. Cystic Fibrosis

Cystic fibrosis (CF) is the most common life-threatening recessive genetic disease in the Caucasian population, affecting approximately 70,000 individuals worldwide, with median predicted life expectancy around the age of 40. This genetic disorder, caused by a mutation of the CF transmembrane conductance regulator (CFTR) gene, is mostly characterized by recurrent lower respiratory infections, exocrine pancreatic insufficiency (85% of patients), and increased electrolyte concentration in sweat. The CFTR gene encodes a member of the ATP-binding cassette transporter superfamily, involved in multidrug resistance. The encoded protein functions as a chloride/carbonate exchange channel, driving anions through different lipid-encased cellular compartments [82].

CFTR resides in many endosomal membranes, trafficking to the epithelial surface and back again; it is localized within lipid rafts and alters membrane lipid composition and in particular ceramide-driven membrane lipid rafts [83, 84]. CFTR mutation can be considered* per se* as a real cause of inflammatory disease and, even in sterile condition, CF fetuses exhibit atrophy or metaplasia and absence of villi. Moreover, the tracheal epithelium in infants and young children exhibits dilated airways with thicker epithelial walls [85, 86]. Human CF fetus allografts of lung small airways into mice with severe combined immunodeficiency (missing lymphocytes and NK cells) stimulate an immediate neutrophil over response and tissue damage, suggesting that innate immunity is hyperactivated even in the absence of infection [87].

It was reported that wild type CFTR, possibly acting as a scavenger, uptakes sphingosine-1-phosphate and the structurally related lipids dihydrosphingosine-1-phosphate and lysophosphatidic acid, thus modulating cell responses to these lipids. In the presence of CFTR, sphingosine-1-phosphate intake increases, thus leaving less ligand available for interaction with sphingosine-1-phosphate receptor and its signaling towards proliferation, migration, and angiogenesis. This would explain the abnormal angiogenesis, responsible for fibrosis, in CF disease, due to a higher availability of sphingosine-1-phosphate and to its stimulation of excessive angiogenesis in response to inflammation [88, 89].

CFTR inhibitors GlyH-101 and CFTRinh172 caused a dose-dependent increase in permeability of the pulmonary or bronchial endothelial monolayer. Increased endothelial sphingosine-1-phosphate, either by exogenous treatment or by endogenous inhibition of its degradation, significantly improved the barrier function in CFTR-inhibited monolayers [90].

In addition to the role of sphingosine-1-phosphate in CF, the most significant aspect of CFTR dysfunction, according to the published papers overall, is an imbalance of sphingolipid homeostasis, due to ceramide release from sphingomyelin within the membranes [91] and to a disease-related increase in ceramide synthesis [83, 92].

A considerable amount of evidence indicates that ceramide may be a pharmacological target in CF, since its accumulation significantly contributes to sustaining hyperinflammation and inability to fight lung infection. The first evidence of lipid and sphingolipid imbalance in CF was reported in the late seventies with the analysis of various lipids, including glycosylated ceramides, in the bronchial lavage and sputum of CF patients [93, 94]. Ceramides, mainly bearing C16:0, C18:0, and C20:0 acyl chains, were shown to accumulate progressively in the lower airway, as the disease advanced, in cystic fibrosis patients, compared with pulmonary hypertension and emphysema patients and healthy donors. Ceramide accumulation correlated with infiltrate presence (neutrophils) [95]. UIrich and coauthors demonstrated extensive inflammation and tissue remodeling in the alveolar tissues from CF patients with advanced lung disease, showing increased myofibroblasts, intercellular adhesion of molecule-1 and collagen expression, and decreased elastin fibers. In these patients alveolar type II cells were markedly stained with anti-ceramide antibodies, demonstrating a close association between inflammation ceramide accumulation and damage within the lower airways [96].

This evidence contrasts with Becker and coworkers' reported data, showing a decrease in ceramides and docosahexaenoic acid (DHA) and an increase in arachidonic acid (AA) content in CF plasma compared with healthy controls. The reduced levels of circulating ceramides, showed by the authors, may be ascribed to CF patients' low plasma HDL, LDL, and total cholesterol levels, due to malabsorption and altered liver function [97]. In these authors' hands, the administration of fenretinide to patients corrected ceramide and other lipids levels [98]. Fenretinide is a known chemotherapeutic agent that modulates sphingolipids by inhibiting a dehydrogenation reaction that forms ceramide, thus accumulating dihydroceramide. Data supporting a mechanism which could explain the plasma ceramide upregulation upon fenretinide treatment are missing. However, fenretinide has different intracellular targets leading to apoptosis that are not related to the inhibition of dihydroceramide desaturation and that may in turn cause ceramide release from apoptosis-related Sphingomyelinases activation [99].

A more thorough knowledge of sphingolipid signaling in CF is obtained from studies in animal models. It is worth noting that both circulating and pulmonary lipid analyses must be obtained only in normal diet fed CF animal models. These can be either low CFTR expressing or CFTR knockout but corrected for wild type CFTR expression in the gut only [83, 100]. In these models, most reports confirm the hypothesis that accumulation of ceramide in the lung promotes apoptosis, DNA deposition, and granulocyte hyperactivity, thus facilitating infection [91].

The alveolar epithelium comprises two main cell types: alveolar type I and alveolar type II cell. Type I cell is a complex branched cell with multiple cytoplasmic plates that are greatly attenuated and relatively devoid of organelles; these plates represent the gas exchange surface in the alveolus. On the other hand, type II cell responds to damage of vulnerable type I cell by dividing and acting as a progenitor cell for both type I and type II cells. In addition, it synthesizes, stores, and releases pulmonary surfactant into the alveolar hypophase, where it acts to optimize conditions for gas exchange [101]. Alveolar spaces are primarily involved in inflammatory responses, being endowed with extended capillary distribution and resident phagocytes. Moreover, alveolar epithelial cells cooperate with alveolar macrophages in immune response and pathogen clearance, by expressing TLRs upon infection [102]. The greatest accumulation of ceramide in CF animals is in the lower airways and, specifically, in alveolar type II cells [96], although a marked accumulation of ceramide was also seen in tracheal and intestinal epithelial cells of low CFTR expressing (solid diet fed) mice [103]. The increased ceramide concentration in alveolar epithelia may significantly contribute to inflammatory signaling and impaired pathogen clearance exerted by these particular cells, addressing the lower airways as the specific therapeutic targets in chronic lung inflammation and infection (Caretti A. and Signorelli P. unpublished data).

Teichgräber et al.'s lab demonstrated an accumulation of ceramide located in intracellular vesicles (not restricted to lysosomes) in CFTR deficient respiratory epithelial cells. According to these authors, CFTR deficiency increases the pH of this vesicular compartment (around pH 6), causing an imbalance between the enzymatic activities of acid Sphingomyelinase and Ceramidase. While acid Sphingomyelinase activity was partially compromised by the pH variation, acid ceramidase activity was dramatically reduced by 90%. Such alteration of activities ensures a release of ceramide from sphingomyelin and then overcomes its clearance by degradation [91]. The same group, based on previously reported data [104, 105], claimed that CFTR deficiency increases the pH of secretory vesicles produced by alveolar macrophages upon* P. aeruginosa* infection, correlating with a reduced production of ROS and reduced bactericidal activity and with ceramide intracellular accumulation and altered lipid raft formation on the plasma membrane [106].

In support of the ion imbalance hypothesis in CFTR deficiency, Noe et al. suggested that increased pH and ceramide are responsible for aberrant angiogenesis, leading to fibrosis in CF disease. Impairment of physiological apoptotic response to stress in the endothelium may lead to abnormal angiogenesis and chronic inflammation. Ceramide is a key regulator of survival and apoptosis in endothelial cells. Oxidative stress, as from hydrogen peroxide, induces ceramide increase mainly via* de novo* sphingolipid synthesis in endothelial cells. Inhibition by CFTR(inh)-172 of endothelial cell channel activity prevented the increases in the ceramide: sphingosine-1-phosphate ratio induced by hydrogen peroxide, impairing caspase activation and apoptosis [107], thus promoting aberrant proliferation under stress and pathological angiogenesis. This evidence would suggest that CFTR deficiency modulates ceramide by altering the pH of intracellular compartments, leading to diverse outcomes according to the cell type.

In line with the hypothesis of an increase in ceramide due to a pH dependent inactivation of acid ceramidase; sphingosine presence on the surface of nasal epithelial cells from CF patients was shown to be almost undetectable (by means of an anti-sphingosine antibody), whereas it is abundantly expressed on the luminal surface of human nasal epithelial cells obtained from healthy individuals. Similar results were obtained in bronchial cells from CF mice. With the aim of demonstrating that sphingosine deficiency may favor bacterial colonization, the inhalation of purified acid ceramidase or sphingosine or its analogue FTY720 was obtained in pulmonary infected CF mice. The treatment not only corrected the anomalous absence of mucosal sphingosine in CF murine airways but also significantly reduced increased ceramide levels and allowed an effective response against lung colonization by* P. aeruginosa*. These results were obtained by treating either prior to or after infection, indicating this therapy as preventive as well as curative against bacterial infection [108]. Although the idea of a reservoir of mucosal sphingosine as a toxic compound, to be spent against pathogens invasion, is attractive, the hypotheses put forward by these authors will certainly require further studies for validation. A major issue that needs to be cleared up, in our opinion, is as follows: given that sphingosine is a toxic compound for eukaryotic cells, how is it possible to obtain beneficial effects in terms of killing bacteria by exogenous administration, avoiding damage to the host?

Although this “pH theory” would fit the above reported published data, by means of CFTR overexpression systems and loss-of-function studies, other investigators reported that neither the secretory (Golgi and TGN) nor the endocytic organelles (endosomes, lysosomes, and phagosomes) display a CFTR-dependent acidification defect (revised in [109]), questioning the technical methods previously used to measure intracellular pH [110]. These latter studies clearly show that CFTR-independent and overall counter-ion permeability was remarkably higher than the passive proton permeability of endosomes, lysosomes, and phagolysosomes of respiratory epithelia and primary or immortalized mouse macrophages. Therefore, CFTR activation cannot interfere with the endosomal pH regulation [109] and ceramide modulation should derive from mechanisms other than pH deregulation of Ceramidase.

In line with the observations arising from their patients' studies, Teichgräber and his coworkers investigated the hypothesis of a hyperactivation of acid Sphingomyelinase in CF animal models. Although the baseline activities of acid Sphingomyelinase and acid Ceramidase in lung homogenates from CFTR deficient animals are equivalent to those in control mice [91], they achieved partial inhibition of the activity of acid Sphingomyelinase either by intraperitoneal injection of amitriptyline or by heterozygous deficiency for acid Sphingomyelinase and observed reduced airway inflammation, phagocyte recruitment, and susceptibility to infection by* Pseudomonas aeruginosa* [91, 111].

Other authors confirm the long term efficacy of amitriptyline to inhibit the robust burst of inflammatory response in CFTR deficient (gut corrected) mice subjected to* Pa*-LPS-induced acute lung injury. In such a model, the authors also demonstrated an early anti-inflammatory activity of fumonisin B1, which is lost at a longer time. Although not explained, the early anti-inflammatory effect of this last inhibitor can be related to its ability to activate Sphingosine Kinase-1 [43, 112, 113] and, therefore, to the upregulation of sphingosine-1-phosphate levels. Moreover we believe that fumonisin B1 can also reduce a burst of ceramide arising from* de novo* synthesis activation in inflammation [92], which in turn sustains the activation of Sphingomyelinase(s) [44]. Nonetheless, the inhibition of Ceramide Synthase by fumonisin B1 is associated with the inevitable mounting, over time, of toxic sphingoid bases. The bioactivity of these metabolites may later on overcome the initial anti-inflammatory action of the inhibitor [114].

CFTR deficiency in mice results in the upregulation of CD95, crucially involved in aseptic inflammation, bronchial cell death rate, and susceptibility to infection. CD95 is activated upon increase of membrane ceramide concentration; CD95 also contributes to stimulating further ceramide release, possibly concurring in the inflammatory pathology of CF [97].

Whereas CF mice exhibit upregulation of inflammasome components and an altered presence of tight junction proteins in lung epithelia, knocking out (heterozygous gene deletion) acid Sphingomyelinase in the same CF mice model resulted in animals similar to the healthy control mice [115]. In addition, CF mice exhibit increased rates of cell death, increased cytokines concentration, and ceramide levels in the trachea and intestine. The inhibition of acid Sphingomyelinase activity ensured the concomitant normalization of cell death, inflammatory cytokines, and ceramide concentration in these body districts [103]. These findings suggest that ceramide plays a crucial role in inflammation and in increased rates of cell death in several organs of cystic fibrosis mice.

The “take home message” derived from the above reported findings is that ceramide and/or its metabolite sphingosine play a crucial role in inflammation and increased rates of cell death in several organs of cystic fibrosis mice [103]. The encouraging results led to the initiation of clinical trials with amitriptyline: the drug was administered to patients 25–50 mg/d twice daily for 28 days. Increased FEV1 and reduced ceramide in nasal and lung cells were observed, with no evident sign of toxicity for the patients [116, 117].

Although ceramide accumulation in CF is clearly established, in our opinion further studies are required in order to consider amitriptyline as a pharmacological tool in CF. The mechanism-based hypothesis of pH induced Ceramidase inactivation and acid Sphingomyelinase hyperactivity is questionable (as above explained) and, to the best of our knowledge, there are no data demonstrating either a transcriptional or a posttranscriptional control of this enzyme* in vivo* in CF or CF models. Data obtained from acid Sphingomyelinase deficient mice are never accompanied by an overview on the sphingolipid metabolism rearrangement in this model nor are they characterized for any possible alteration in their immune responses. The reported quantitations of ceramide increase in CF mice versus wild type are also puzzling, with an approximately 8-fold increase when measured by DAG Kinase total ceramide phosphorylation and Thin Layer Chromatography separation [111, 115], compared with mass spectrometry analytical methods (around 20–30% increase) [92, 100]. Prolonged treatment with amitriptyline (6 months) was performed on CF mice, monitoring the reduced level of ceramide, but there are no data on the response to infection and on the overall immune response of the treated animals [111]. Finally, the requirement for acid Sphingomyelinase activation in macrophages and immune cells (i.e., ROS production and bactericidal activity [106]), to give rise to proper signaling during infection, leaves an open question as to the efficacy of this treatment in patients with chronic infections.

An alternative explanation of sphingolipid metabolite imbalance in CF is derived from studies conducted by Wargall's group and our own. It relies on the hypothesis that CFTR deficiency induces intracellular stress leading to an upregulation of the rate-limiting step of sphingolipid* de novo* synthesis, with consequent accumulation of ceramide. Since an intracellular increase of ceramide stimulates Sphingomyelinases (as effectively as TNF*α*), thereby amplifying the Sphingomyelinase activation [44], this mechanism may also explain the effect of amitriptyline administration in CF models. By means of radioactive precursor treatment (^3^H serine and ^3^H sphinganine), elegant metabolism studies revealed that sphingolipid synthesis is significantly enhanced in IB3 CF epithelial cells compared to normal C38 cells: increased rate of radioactivity incorporation was assessed, for* de novo* as well as the recycle path of ceramide synthesis and for sphingomyelin synthesis. Moreover, a markedly enhanced expression of Serine Palmitoyl Transferase 1 subunit was found in CF cells, and its expression inversely correlates with CFTR expression in airway epithelial cells. The mass of C16-dihydroceramide and C22- and C24-ceramide species increased compared with controls, whereas C18-ceramide and C18:1-ceramides mass decreased [83]. Similar results were obtained by our group comparing the same cell lines under inflammatory stimulation: we concluded that TNF*α* was able to significantly enhance Serine Palmitoyl Transferase 1 transcript in IB3 cells and that inflammatory cytokines transcription and release, induced by TNF*α*, were impaired by treating cells with myriocin [92], the inhibitor of Serine Palmitoyl Transferase (the rate-limiting enzyme in the sphingolipid* de novo* synthesis pathway [13]). Next, we demonstrated that the response against* P. aeruginosa* acute lung infection in CF mice is ameliorated by intratracheal treatment with myriocin and that both ceramide and inflammatory mediators pulmonary levels of these mice were corrected to those of wild type mice [92].

Although enhanced sphingolipid synthesis in CF was shown to induce ceramide and sphingomyelin mass increase, the CFTR silenced human airway epithelial cell line exhibited ~60% lower GM1 ganglioside than control cells and was unable to migrate, showing impaired activation of *β*1-integrin, phosphorylation of focal adhesion kinase, and Crk-associated substrate. Exogenously added GM1 partially restored migration of CFTR silenced cells [118]. This deficiency in gangliosides may be related to dysregulated intracellular trafficking of neosynthesized ceramide and may account for reduced antibacterial responses, since gangliosides are involved in bacteria interaction (as previously mentioned). It should be noted that the loss of CFTR function leads to altered cholesterol trafficking, resulting in increased cholesterol synthesis. Excessive cholesterol causes pathological conditions such as atherosclerosis. It is recognized that sterols can modulate the levels of other lipids to attain lipid homeostasis; thus, excess free cholesterol may play a role in modulating compensatory sphingolipid pathways [119] and it is suggested that this perturbation in cholesterol regulation contributes to the inflammatory response present in CF [120].

The overall data suggest that decreased CFTR expression would reflect a state in which augmented membrane lipid synthesis, including sphingolipids, is necessary to compensate the dysfunction and maximally increase membrane stability. This hyperanabolism state either associates with or possibly promotes hyperinflammation, with ceramide being a major signaling mediator. This concept is further sustained in the following paragraph, dealing with sphingolipids involvement in COPD pulmonary inflammation [83].

**Figure 2:**
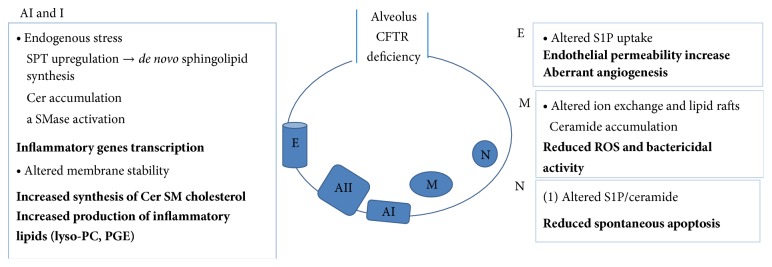
Sphingolipids modulation and bioactivity in cystic fibrosis: prevalent sphingolipids alteration in the alveolar lung compartment and induced responses in endothelial cells (E), alveolar epithelial cells (alveolar type 1 AI, alveolar type 2, AII), macrophages (M), and neutrophils (N). SPT, Serine Palmitoyl Transferase; Cer, ceramide; a SMase, acid Sphingomyelinase; SM, sphingomyelin; lyso-PC, lysophosphatidyl choline; PGE, prostaglandin; S1P, sphingosine-1-phosphate; ROS, reactive oxygen species.

The evidence discussed above is summarized in Figure 2.

### 5.3. Chronic Obstructive Pulmonary Disease

Chronic obstructive pulmonary disease (COPD) is an inflammatory respiratory disease, estimated to become the third leading cause of death worldwide by 2020, after ischemic heart disease and cerebrovascular disease [121, 122]. The Global Initiative for Chronic Obstructive Lung Disease (GOLD) defines COPD as follows: COPD, a common preventable and treatable disease, is characterized by airflow limitation that is usually progressive and associated with an enhanced chronic inflammatory response in the airways and the lung to noxious particles or gases. Exacerbations and comorbidities contribute to the overall severity in individual patients (global strategy for the diagnosis, management, and prevention of chronic obstructive pulmonary disease: revised 2014; Global Initiative for Chronic Obstructive Lung Disease (GOLD), available online: http://www.goldcopd.org/). Salvi and Barnes, from population-based studies, proposed that cigarette smoke is a risk factor only for approximately half of COPD patients, and alternative genetic and epigenetic environmental risk factors are clearly implicated in the disease's etiology. The triggering causes of COPD include indoor and outdoor air pollution (i.e., biomass fuel, dust, and fumes [123]). A central role in the pathophysiology of COPD has been shown to be played by chronic inflammation of the airways [124]. Although cause related, the inflammation process characterizing COPD persists long after cessation of stress (i.e., quit smoking) and relies on cells and mediators ranging from innate to adaptive immunity, ROS overproduction and induced damage, imbalance of local proteolysis/antiproteolysis, fibrosis, and altered angiogenesis. The outcome is a thickened (and also dysfunctional) epithelial layer, increased thickness of the smooth muscle layer of the airway, disruption of the alveolar walls with varying levels of fibrosis, hypoxic vasoconstriction [125], the narrowing and progressive loss of terminal bronchioles and increased peripheral airway resistance, and arterial hypertension [126]: all these features precede emphysematous demise of the COPD lung structure [127]. Depending on the patients (age, susceptibility to infection, etc.), hyperinflammation is conducive to an increase in the number and size of mucus-secreting glands. Such condition, named mucus metaplasia, causes mucus hypersecretion, increased mucin stores in the epithelium according to airflow limitations, and increased luminal mucus obstructing the airways and evolves into chronic bronchitis [128]. Thus chronic bronchitis and emphysema often overlap in COPD patients, with the burden of one or the other prevailing. Susceptibility to exacerbations is defined by background inflammation in the lung tissue, microbiota equilibrium, and comorbidities. Individual immune responsive ability and autoimmunity have been indicated as possible initiators of the pathologic inflammation of COPD [129]. Up to the 1960s, the imbalance between proteases and antiproteases induced during inflammation was ascribed as the major mechanism initiating emphysema and COPD pathogenesis. In the last decade, however, the collapsing of the alveolar structure was explained in terms of apoptosis of epithelial and endothelial cells and of excessive inflammatory stress. Interestingly, autophagy was demonstrated to be significantly impaired, with marked accumulation of p62-enriched vesicles in the epithelia of COPD patients, accounting for accumulation of damaged material and unbalanced homeostasis between degradation and resynthesis [130, 131].

In line with this evidence, metabolic changes responsible for proinflammatory metabolites accumulation can be considered as one of the triggering causes concurring in COPD etiology: specifically, sphingolipid mediators contribute to the inflammatory process, driving the onset and progression of the pathology.

In 2005, Petrache and colleagues induced emphysema in mouse lung by subcutaneous treatment with the VEGF receptor 1 and receptor 2 inhibitor, SU5416. They demonstrated that apoptosis was mediated by ceramide, whose accumulation was localized at the alveolar septal cells and not in bronchi, colocalized with caspase-3 activation and most importantly preceded (by about twenty days) alveolar enlarging and damage. The accumulation of ceramide depended on its* de novo* synthesis and the authors were able to prevent VEGFRs inhibitor-induced damage by systemic administration of fumonisin B1 or myriocin. Indeed, a later activation of acid Sphingomyelinase was found in its secreted form, released as a feed-forward mechanism in response to enhanced sphingolipids synthesis, thus increasing the pool of paracellular ceramide and amplifying lung inflammation and injury. Anti-ceramide antibody i.p. administration was partially able to neutralize extracellular ceramides and attenuated lung apoptosis, induced by the VEGFR inhibitor. To better prove that the altered sphingolipid metabolism severely affects lung physiology, they administered fumonisin B1 to untreated normal mice and observed alveolar enlargement and damage, which was inhibited by replenishment of sphingoid bases (sphingosine-1-phosphate and FTY720 combinatorial treatment, with the aim of reintroducing sphingoid bases but simultaneously downregulating the sphingosine-1-phosphate receptor 1 signaling). FTY720 and exogenous sphingosine exert a protective effect on airspace enlargement, concomitant with attenuation of VEGFR inhibitor-induced lung apoptosis, possibly by decreasing the ceramide/sphingosine-1-phosphate ratio (by FTY720 inhibition of Ceramide Synthesis and induction of Sphingosine Kinase, resp.) [43]. Moreover, intratracheal instillation of synthetic ceramide analogue C12 was able to induce septal apoptosis and emphysema, by triggering the synthesis of long-chain endogenous ceramide, which accumulated twofold [44]. As alveolar cell apoptosis and oxidative stress mutually interact to mediate alveolar destruction [39], the same research group demonstrated, shortly afterwards, that intratracheal ceramide instillation decreased cytosolic SOD activity and increased superoxide production in the lungs. They also demonstrated that overexpressing human Cu/Zn SOD in mice significantly protected from intratracheal ceramide-induced superoxide production, apoptosis, and air space enlargement. It is to be noted that the lung activation of acid Sphingomyelinase, in response to ceramide treatment, was abolished when overexpressing SOD. Such evidence demonstrates that exogenous ceramide treatment and the consequently stimulated endogenous neosynthesized ceramide merge in ROS formation and acid Sphingomyelinase activation. This latter activity amplifies injury through redox-dependent mechanism. Both ROS accumulation and acid Sphingomyelinase derived ceramide are the major effectors of the damage, leading to the conclusion that enhanced neosynthesis of lung ceramides is upstream of redox-dependent inflammatory damages [132]. Knowing that cigarette smoke induces oxidative stress and is one of the causes of emphysema, mass spectrometry analysis proved that cigarette smoke is able to induce ceramide accumulation in the murine lung [132]. Accordingly, lung ceramide levels were markedly higher in patients with emphysema due to chronic cigarette smoking compared with patients without emphysema, as measured by Diacylglycerol Kinase assay, followed by Thin Layer Chromatography and ceramide quantitation. Immunohistochemistry revealed that ceramide was almost exclusively localized to alveolar septal cells and alveolar macrophages. Human emphysematous lungs exhibit a significant increase in long-chain ceramides. Even smoking alone (without a pathological diagnosis of emphysema) changed the lung ceramide expression profile, suggesting that not only absolute levels but also patterns of ceramide species expression may be upregulated and contribute to emphysema induction. These studies highlight the concept that ceramide accumulates in specific lung compartment and there induces damage in epithelial cells, which in turn supports inflammation in a vicious loop. Ceramide accumulation results from deregulation of sphingolipid metabolism. The accumulation of newly synthesized sphingolipids (bearing long acyl chains) leads to the extracellular release of ceramide(s). In addition, an increased synthesis of catabolism enzymes, such as Sphingomyelinases, is required in response to the sphingolipids accumulation, and eventually the extracellular release of these enzymes occurs too. These events are linked to prolonged extracellular inflammatory/damaging signaling [44]. Levy and colleagues substantiated the hypothesis that cigarette smoke induces the activation of neutral Sphingomyelinase 2 and apoptosis in human respiratory epithelial cells and glutathione administration was able to inhibit apoptosis [133]. Mice exposed to cigarette smoke exhibit a twofold increase in lung ceramide (measured by Diacylglycerol Kinase assay) as well as a significant increase in neutral Sphingomyelinase expression (evaluated by immune histochemical staining of the protein with noncommercial antibodies), both in the bronchial epithelium and in alveolar septal cells. Diet supplementation with N-acetyl-cysteine (glutathione precursor), or intranasal installation of biotin-labeled neutral Sphingomyelinase 2 siRNA, showed a significant reduction in this enzyme staining and of lung apoptosis, measured both by TUNEL assay and by evaluation of cleaved caspase-3 levels. Immunostaining for neutral Sphingomyelinase 2 on lung biopsies from emphysematous smoker patients versus healthy people showed an enhanced expression of the enzyme [134]. Finally, the oncogene product Src Kinase was shown to activate the serine kinase p38, responsible for phosphorylation and activation of neutral Sphingomyelinase 2, upon oxidative stress [135].

The catabolism of ceramide gives rise not only to sphingosine but also to the opposing bioactive sphingosine-1-phosphate, known to promote proliferation and survival, even though in very low intra- or extracellular concentration [43]. Systemic administration of sphingosine, via daily intraperitoneal injection, increased sphingosine-1-phosphate levels in a dose-dependent manner. In the presence of VEGFR blockade, stimulation of sphingosine-1-phosphate activity, by either administering its precursor D-erythro-sphingosine or the agonist of its receptor 1, SEW2871, led to a decrease in the ceramide to sphingosine-1-phosphate ratio and markedly reduced apoptosis and lung injury [43].

Sphingosine-1-phosphate receptors 1–3 (S1PRs) are involved in the control of pulmonary vascular function by altering endothelial and epithelial barriers as well as smooth muscle cell function [48, 136, 137]. In mice, S1PR3 receptor activation induced pulmonary edema by opening narrow junctions between alveolar type I and type II cells [137], and S1PR2 deficient mice and mice with reduced S1P3 receptor expression were protected against LPS-induced disruption of the alveolar barrier [136, 137]. A recent report, analyzing the expression of the genes related to sphingosine-1-phosphate synthesis/degradation and of its receptors in human lung, showed that relative mRNA expression of S1PR5 was significantly reduced in COPD patients compared with control [138]. A possible scenario in which different S1PRs are differentially modulated in the airways to promote inflammation can be envisaged from these reports.

Environmental stresses, including cigarette smoke and hypoxia, and chronic inflammation have recently been shown to reduce CFTR function, and this suggests that common mechanisms contribute to the progression of both CF and COPD [114]. Immune histochemical analysis of tissue sections reveals that CFTR expression is inversely correlated with the severity of emphysema and with ceramide accumulation in COPD subjects, compared with control subjects [84]. Bodas and colleagues also demonstrated that acute exposure to cigarette smoke induces a significant downregulation of CFTR in lung and in particular in membrane rafts. From antibody staining, they claim that the absence of CFTR in rafts induces the accumulation of ceramides within the membrane, possibly as a compensatory mechanism for the altered stability of the membrane [84]. Although it is risky to draw conclusions on ceramide levels from quantitation obtained by staining with anti-ceramide antibodies, these data are in line with other published results, which suggest that deficiency of CFTR switches on an “alarm” mechanism that leads the cell to modulate the synthesis of membrane components [83]. In this view, an upregulation of sphingolipid synthesis not only fulfills the requirement for supporting raft anomalies but also triggers an alarm signal that evolves toward a chronic inflammatory state.

Treatment with CFTR inhibitors GlyH-101 and CFTR(inh)-172 caused a dose-dependent increase in human and rat pulmonary endothelial monolayer permeability, redistribution of the junctional protein *β*-catenin, and scattered actin stress fiber formation. Endothelial cells treated with exogenous sphingosine-1-phosphate or with endogenous sphingosine-1-phosphate lyase inhibitor exhibited a significantly smaller decrease in permeability in response to CFTR inhibition. In this model, exposure to cigarette smoke markedly enhanced the loss of endothelial barrier function [90].

To the degree that the catabolism of ceramide gives rise to sphingoid bases and these become phosphorylated to produce sphingosine-1-phosphate, the metabolism of ceramide via direct phosphorylation forms ceramide-1-phosphate, another metabolite opposing ceramide and signaling towards proliferation and survival. In a recent publication, ceramide-1-phosphate (bearing either the short chain analogous C8 acyl chain or the C16 natural acyl chain) was administrated intratrachea. The administration occurred after acute cigarette smoke inflammation or it lasted for the last three months, overlapping with the last part of a seven-month cigarette smoke treatment (long treatment to model chronic inflammation). Ceramide-1-phosphate significantly reduced inflammation (cytokines lung expression and BAL infiltrate) as well as alleviating lung emphysema occurring after chronic stress [52]. Ceramide-1-phosphate effects were recapitulated* in vitro*, blocking cigarette smoke induced hyperinflammation in human airway epithelial cells and neutrophils from COPD patients [52].

It is intriguing to note that the above reported data suggest that ceramide-1-phosphate and sphingosine-1-phosphate, known to modulate inflammation with different mechanisms and outcomes, may be applied in pulmonary chronic inflammatory disease with a mere anti-inflammatory action. Our hypothesis is that, in stress conditions such as chronic inflammation, the ER major and ubiquitous cellular sensors stimulate* de novo* synthesis of ceramides as an intrinsic response to the stress, affecting the whole network of the sphingolipid metabolism, leading to membrane reorganization and signaling modulation. In order to balance the increased signaling of ceramide, its opposite metabolites ceramide-1-phosphate and sphingosine-1-phosphate, even if present at lower concentrations than the ceramide, may be able to counteract ceramide-induced outcomes. Thus the bioactivity of ceramide should rather be considered not* per se* but as a ceramide/ceramide-1-phosphate or ceramide/sphingosine-1-phosphate ratio. It is also necessary to take into account the possibility of ceramide-forming sphingosine, which can be considered as proinflammatory “lyso-ceramides,” in opposition to forming sphingomyelin and glycosphingolipids. This complex picture might be envisaged for therapeutic approaches, although requiring much more thorough translational studies.

Lung chronic disease is to be considered as a systemic inflammatory disease and medical research focuses on the identification of early new markers in blood and body fluids [139, 140]. A massive presence of neutrophilic infiltrate along with increased inflammatory mediators signaling (i.e., IL-6) has been detected in the sputum of patients with COPD [141] as well as in bronchoalveolar lavage fluid (BALF) [142]. Bahr and colleagues studied the peripheral blood mononuclear cells expression profile in 136 subjects of the COPD gene cohort. Among others they found an overexpression of acid ceramidase in COPD and emphysema patients but not in those with chronic bronchitis. Looking for metabolites, to validate the gene expression results, they found an accumulation of lactosyl ceramide. The authors did not clearly explain the link between the enzyme and the metabolite [143]; anyhow these evidences confirm the spread of inflammation. One year later, the analysis of sputum samples demonstrated that there was an increase in different sphingolipids species (ceramides, sphingomyelin, and gangliosides) in smoking compared with nonsmoking COPD patients. Specifically, ceramide concentration is inversely related to the severity of the disease. A part of these sphingolipids was significantly reduced upon cessation of smoking [144]. A hypothesis for these apparently contrasting data was launched in a recent large-scale study of more than 250 subjects from the COPD gene cohort, demonstrating that plasma sphingomyelin, ceramide, ganglioside GM3, monohexosyl ceramide, and sphingosine-1-phosphate levels inversely correlated with COPD worsening phenotype (low FEV1) and emphysema, whereas trihexosylceramides correlated directly with exacerbations. In agreement with previous data from peripheral blood mononuclear cells, no correlation with chronic bronchitis phenotype was traced. Plasma sphingolipids may derive from shed cellular plasma membrane, apoptotic bodies, microvesicles, or exosomes. The decreased levels of sphingomyelin in plasma were ascribed to increased catabolism activities by enhanced/activated plasma secreted acid Sphingomyelinase and neutral Ceramidase [74, 145]. These data suggest that, in severe COPD and its exacerbations, plasma accumulation of sphingolipid catabolites and related secreted enzymes possibly contributes to systemic inflammation [146].

An impairment of apoptotic cell phagocytosis in emphysema lungs, leading to secondary necrosis and promoting inflammation, has been reported [147, 148]. Being a regulator of autophagy, ceramide lung enrichment may interfere with autophagosomes clearance. Intratracheal instillation of ceramide (PEG-C16-ceramide) after intrapulmonary introduction of apoptotic (PI-targeted) human thymocytes significantly decreased the phagocytic uptake of the exogenous cells by alveolar macrophages, recovered from BAL [149]. The authors conclude that, upon occurrence of inflammatory events, such as the exogenous ceramide instillation, endogenous ceramide accumulates and gives rise to sphingoid bases that are primarily responsible for phagocytosis inactivation: alveolar macrophages recovered their phagocytic ability, impaired by cigarette smoke extracts, when pretreated with myriocin (to inhibit* de novo* synthesis of sphingolipids) or MAPP or siRNA to inhibit acid ceramidase but not with fumonisin B1 (to inhibit sphingosine acylation) nor Sphingomyelinase inhibitors [149]. Moreover, the sphingosine intracellular increase caused by exposure to cigarette smoke was responsible for an altered lipid raft assembling, impairing the presence in the membrane of Rac1 GTPases, which promotes membrane ruffling and phagosome closure [149].

Emphysema is caused by alveolar structure demise. This destructive process particularly targets lung microvascular endothelial cells and alveolar epithelial cells. Besides apoptosis, aberrant lung endothelial cell responses may contribute to pulmonary vascular remodeling, frequently observed in COPD in response to the low oxygen level caused by terminal bronchiolar loss, favoring endothelia permeability and inflammation [150, 151]. Petrache's group conducted an interesting study using primary human lung microvascular endothelial cells derived from smokers or nonsmoking healthy subjects. Following the hypothesis that ceramide synthesis is increased upon exposure to smoke and that this ceramide is released and acts paracellularly, they treated endothelial cells with palmitoyl ceramide (Cer16). While cells from nonsmokers responded with apoptosis, the smoker-derived cells were found to be resistant to exogenous ceramide and exhibited marked proliferation and increased autophagy. This involved a baseline-increased phosphorylation of Akt (a known prosurvival kinase, inhibited by ceramide and possibly enhanced as a feed-forward mechanism in an environment with baseline-increased ceramide) and increased levels of high-mobility group box 1 (an inflammatory protein, elevated in sputum and plasma of COPD patients and directly involved in vasculature remodeling in emphysema [150]). The authors hypothesize that deregulated sphingolipid metabolism and increased endogenous sphingosine, arising from increased ceramide production, may contribute to inflammation by promoting the expression and the activity of high-mobility group box 1 and aberrant vasculogenesis.

A correlation between lactosyl ceramide accumulation and increasing severity of emphysema was found in lung tissues from COPD subjects and in cigarette smoke treated mice lungs. The increase in this sphingolipid was impaired by pharmacological inhibition of lactosyl Ceramide Synthase. Moreover, in bronchial-epithelial cells (BEAS2B) and macrophages (Raw264.7), lactosyl Ceramide Synthase inhibitors impaired aberrant-autophagy (p62-accumulation) and apoptosis induced by cigarette smoke extracts [152].

**Figure 3:**
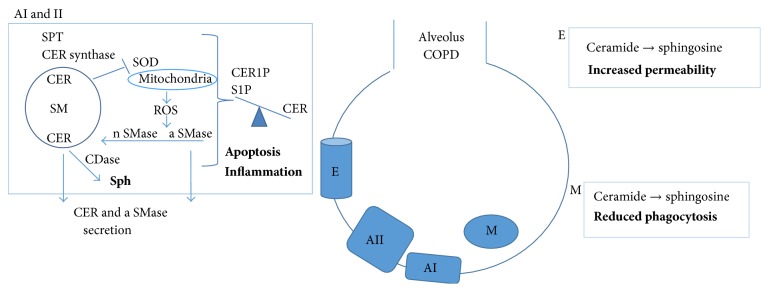
Sphingolipids modulation and bioactivity in COPD: prevalent sphingolipids alteration in the alveolar lung compartment and induced responses in endothelial cells (E), alveolar epithelial cells (alveolar type 1 AI, alveolar type 2, AII), and macrophages (M). SPT, Serine Palmitoyl Transferase; Cer, ceramide; SM, sphingomyelin; Sph, sphingosine; a SMase, acid Sphingomyelinase; n SMase, neutral Sphingomyelinase; CDase, Ceramidase; S1P, sphingosine-1-phosphate; Cer1P, ceramide-1-phosphate; ROS, reactive oxygen species.

Overall, the evidence cited above (summarized in Figure 3) traces an important role for ceramide synthesis and its derived metabolites in sustaining chronic lung inflammation.

## 6. Conclusions

Sphingolipid molecules play an active part in the extracellular equilibrium of proinflammatory and anti-inflammatory lipids which finely regulate mucosal activities and immunity in the lungs. In addition to being structural membrane components and endogenous mediators, sphingolipids exert an additional paracrine signaling role which is required for physiological immune response, defense from pathogens, and inflammation resolution. Deregulation of sphingolipid mediator homeostasis alters the development of the fetal lung and impairs its maturation and functionality. In adult life, deregulation of sphingolipid mediators is associated with a derangement of the inflammatory cascade. As outlined above, a major distinction can be traced between the roles of sphingolipids in acute and chronic lung inflammation. Acute inflammation is triggered by a robust and sudden stress, which sets off the rapid formation of alarm signals. The hydrolysis of membrane sphingomyelin is primarily involved in this external incident-intracellular response setting. Local ceramide increase produced by Sphingomyelinases activates different pathways cooperating in the activation of immune defense, cell stress response, and eventually apoptosis in case of cell failure. Chronic inflammation, on the other hand, is* per se* the cause of a prolonged hidden and borderline stress, which alters cell features and eventually tissue physiology and function. In this scenario, as a consequence of either a genetic defect or environmental toxic factors such as pollutants, the whole sphingolipid metabolic network undergoes a stable shift requiring transcriptional regulation. An overall increase in sphingolipid synthesis, coordinated with that of other lipids devoted to forming surfactant, reflects an attempt to enhance mucosal protection. This is a fragile equilibrium that may be compromised if an additional external insult, such as an infection, supervenes; the equilibrium can then collapse into a self-sustaining hyperinflammatory reaction, and the upregulation of the metabolic rate itself turns out to be a pathogenetic rather than protective adjustment. Insofar as this picture is correct, the goal of future translational research must be to obtain a preventive, disease-personalized medicine, targeting the alteration of metabolism induced by the disease. Within the lung, sphingolipid metabolism may constitute a key target because sphingolipid synthesis and ceramide levels seem to control the synthesis of other lipid mediators involved in inflammation, and ceramide modulatory agents have a profound biological effect in processes such as epithelial apoptosis, endothelial permeability and immune cells mobilization, mucus production, and pathogen clearance.

## Abbreviations

PG: Prostaglandin
PAF: Platelet activating factor
TNF: Tumor necrosis factor
ROS: Reactive oxygen species
NO: Nitric oxide.
